# Fibrocytes are associated with vascular and parenchymal remodelling in patients with obliterative bronchiolitis

**DOI:** 10.1186/1465-9921-10-103

**Published:** 2009-10-30

**Authors:** Annika Andersson-Sjöland, Jonas S Erjefält, Leif Bjermer, Leif Eriksson, Gunilla Westergren-Thorsson

**Affiliations:** 1 Division of Medicine and Allergology, Department of Clinical Medical Science, Lund University, Sweden; 2 Division of Airway Inflammation and Immunology, Department of Experimental Medical Science, Lund University, Sweden; 3 Unit of Lung Biology, Department of Clinical Medical Science, Lund University, Sweden

## Abstract

**Background:**

The aim of the present study was to explore the occurrence of fibrocytes in tissue and to investigate whether the appearance of fibrocytes may be linked to structural changes of the parenchyme and vasculature in the lungs of patients with obliterative bronchiolitis (OB) following lung or bone marrow transplantation.

**Methods:**

Identification of parenchyme, vasculature, and fibrocytes was done by histological methods in lung tissue from bone marrow or lung-transplanted patients with obliterative bronchiolitis, and from controls.

**Results:**

The transplanted patients had significantly higher amounts of tissue in the alveolar parenchyme (46.5 ± 17.6%) than the controls (21.7 ± 7.6%) (p < 0.05). The patients also had significantly increased numbers of fibrocytes identified by CXCR4/prolyl4-hydroxylase, CD45R0/prolyl4-hydroxylase, and CD34/prolyl4-hydroxylase compared to the controls (p < 0.01). There was a correlation between the number of fibrocytes and the area of alveolar parenchyma; CXCR4/prolyl 4-hydroxylase (p < 0.01), CD45R0/prolyl 4-hydroxylase (p < 0.05) and CD34/prolyl 4-hydroxylase (p < 0.05). In the pulmonary vessels, there was an increase in the endothelial layer in patients (0.31 ± 0.13%) relative to the controls (0.037 ± 0.02%) (p < 0.01). There was a significant correlation between the number of fibrocytes and the total area of the endothelial layer CXCR4/prolyl 4-hydroxylase (p < 0.001), CD45R0/prolyl 4-hydroxylase (p < 0.001) and CD34/prolyl 4-hydroxylase (p < 0.01). The percent areas of the lumen of the vessels were significant (p < 0.001) enlarged in the patient with OB compared to the controls. There was also a correlation between total area of the lumen and number of fibrocytes, CXCR4/prolyl 4-hydroxylase (p < 0.01), CD45R0/prolyl 4-hydroxylase (p < 0.001) and CD34/prolyl 4-hydroxylase (p < 0.01).

**Conclusion:**

Our results indicate that fibrocytes are associated with pathological remodelling processes in patients with OB and that tissue fibrocytes might be a useful biomarker in these processes.

## Introduction

Lung transplantation (LTP) represents a treatment option in many end-stage lung diseases such as chronic obstructive lung disease, cystic fibrosis, and idiopathic pulmonary fibrosis (IPF). Unfortunately, as many as 60% of patients who have been transplanted develop obliterative bronchiolitis (OB) which is a form of chronic rejection, within 5 years [1]. It is also known that bone marrow transplantation (BMT) after, for example, acute and chronic leukaemia, anaemia, and rare immunodeficiency disorders, can also lead to OB at a frequency of 2-11% [2-4], with similar pathological characteristics as after LTP. The reason that some patients develop OB is still unclear, but one of the most frequent risk factors is repeated acute rejection, followed by lymphocytic bronchitis or bronchiolitis [5]. The histological findings are described as epithelial injury, with mononuclear inflammation and fibrotic lesions that lead to intraluminal polypoid plugs of granulation tissue within the terminal and respiratory bronchioles. The lesions consist of fibroblasts and/or myofibroblasts and extracellular matrix (ECM). The main producers of ECM are lung fibroblasts and phenotypes derived from fibroblasts. There are three current hypotheses concerning the origin of these cells: *1.) *proliferation and/or differentiation of resident fibroblasts [6]; *2.) *epithelial mesenchymal transition (EMT) [7-9]; and *3.) *recruitment of circulating progenitors such as fibrocytes to the lung, where they differentiate further into specific fibroblast phenotypes. Fibrocytes are identified by specific combinations of mesenchymal markers such as prolyl 4-hydroxylase and α-smooth muscle actin (αSMA), together with haematopoietic markers such as CD34, leukocyte markers such as CD45 [10,11], and chemokine receptors such as CXCR4 [12].

In a previous study, our group showed that there is accumulation of sub-epithelial fibrocytes in patients with mild asthma [13]. There was a positive correlation between the number of fibrocytes and the thickness of the lamina reticularis layer of the basement membrane, which is also a characteristic feature of asthma. Furthermore, fibrocytes have been observed near fibroblastic foci in patients with IPF [14]. Bröcker *et a. *also identified recipient-derived αSMA-positive cells in lung tissue in a study of two patients with OB after bone marrow transplantation [15]. When fibrocytes move from the circulation to the injured lung tissue, there is a gradual loss of haematopoietic markers while the expression of mesenchymal markers increases [16].

It has been shown that LTP patients have a reduced number of blood vessels in the small airways before developing obliteration of the airway [17], and that LTP patients with BOS (bronchiolitis obliterans syndrome) have higher microvascular density in endobronchial biopsies than controls. The changed composition of the vessels has been hypothesised to be a tissue reaction to relative hypoxia and hypercarbia [18].

Together, the previous findings have led to the hypothesis that fibrocytes may be an additional source of mesenchymal cells in fibrotic lesions. If so, fibrocytes either circulating or in tissue, could be a possible biomarker for remodelling in OB, and the vascular remodelling in these patients might facilitate an increased influx of fibrocytes into the tissue. These events may play an important role in the development of fibrosis in OB. Hereby we show that the number of fibrocytes identified in tissue was correlated to structural remodelling of both the pulmonary vessels and the alveolar parenchyma.

## Materials and methods

### Patients

The OB patients included in the study had all undergone LTP or BMT (Table 1). The mean age at transplantation was 34.4 years (range: 10-65). The LTP patients were transplanted because of bronchiolitis, emphysema, or cystic fibrosis while the BMT patients had aplastic anaemia or lymphatic leukaemia. The patient group consisted of two females and four males. The study was performed on autopsies from this group of patients and tissues were obtained from part of the peripheral lungs. Control lung tissue was collected during surgery from patients with suspicion of lung cancer but with otherwise healthy lungs (mean age of 65.3 years; range: 50-73). The tissue in the control patients was obtained by wedge resections to make the samples as comparable as possible. Only patients with well-delineated tumours were included and (as far as possible) the tissue was collected possible away from the tumour. None of the controls had ever smoked, and there were four females and two males. This procedure has often been used to collect human control tissue [19]. The study was approved by the local research ethics committee (534/2005).

**Table 1:** Patient information

LTP/BMT	Underlying disease	Age at TP (years)	Time from TP to autopsy (years)
LTP	Bronchiolitis, RA	51	9
LTP	Emphysema	41	6
LTP	CF	21	3
LTP	Emphysema	65	0.5
LTP	COPD	61	1.5
BMT	Lymphatic leukaemia	10	2
BMT	Aplastic anaemia	15	15
BMT	Anaplastic lymphoma	11	8

LTP = lung transplantation; BMT = bone marrow transplantation; TP = transplantation; RA = rheumatoid arthritis; CF = cystic fibrosis; COPD = chronic obstructive pulmonary fibrosis.

### Tissue Preparation and General Histopathological Evaluation

After excision, the tissue was fixed in standard 4% buffered formalin. After dehydration, it was embedded in paraffin. For a gross histological characterization, tissues from both the patient group and the control group were stained with Gomori's trichrome. All analyses were done blinded and also counted by a second person.

### Immunofluorescence of Fibrocytes

After heat-induced antigen retrieval, non-specific binding in lung sections was blocked with Tris-buffered saline (TBS) containing 0.5% BSA and 5% horse serum, and avidin/biotin block according to the instructions of the manufacturer (Vector Laboratories Inc., Burlingame, CA). The sections were incubated overnight with either CXCR4 (R&D Systems, Minneapolis, MN) (15 μg/ml), CD45R0 (5 μg/ml), or CD34 (5 μg/ml). (the latter two from BD Biosciences, Pharmingen, Leiden, the Netherlands). The signals from CD34, CD45R0, and CXCR4 were amplified using biotinylated antibody (1:100 dilution), (Vector Laboratories Inc. Burlingame CA) followed by biotinylated anti-immunoglobulin antibody (1:200 dilution in PBS containing 1% BSA). The specimens were also incubated with primary antibody to prolyl 4-hydroxylase (Acris Antibodies, Hiddenhausen, Germany) (1 μg/ml), and then with a corresponding secondary antibody conjugated with Alexa fluorocrome (Molecular Probes, Eugene, OR), in TBS containing 1% goat serum (Vector Laboratories) for 1 hour. For the combination of CD34 and prolyl 4-hydroxylase, the primary antibody to prolyl 4-hydroxylase was pre-labelled with Alexa fluorochrome according to the instructions included in the Zenon Labeling Kit (Molecular Probes). The nuclei were visualised with DAPI (Invitrogen Corp., Carlsbad, CA). To quantify lung fibrocytes, a field with six randomly selected areas of 0.28 mm^2 ^was analyzed for double-stained cells (i.e. fibrocytes).

### Immunofluorescence of Vessels

Five-micrometer-thick paraffin sections of formalin-fixed lung tissue were incubated overnight with primary antibody to von Willebrand factor (Dako, Glostrup, Denmark) diluted 1:600 in TBS containing 1% BSA. The primary antibody was detected with a secondary antibody conjugated with Alexa-555 fluorochrome (Molecular Probes, Eugene, OR) in TBS containing 1% goat serum (Vector Laboratories) for 1 hour. The nuclei were visualised with DAPI.

### Calculation of Areas and Numbers of Vessels

50 pulmonary vessels with a luminal area of between 400 and 180,000 μm^2^, located more than 500 μm from the nearest bronchiole, were randomly chosen and analyzed for luminal area and thickness of endothelial layer. This was performed by computerized image analysis using the NIS-Elements AR 3.0 system (Nikon) a Nikon Eclips 80i microscope, and a Nikon DS-Qi1Mc camera. Endothelial layer thickness was determined by measuring the thickness of vonWillebrand factor positive endothelial cells. To obtain the lung density of parenchymal pulmonary vessels (defined as being more than 500 μm from a bronchiole), two parameters were used: 1.) the percentage of total lung tissue that comprised luminal area of pulmonary vessel, and 2.) the percentage of total lung tissue that consisted of pulmonary vessel endothelium. Both of these parameters were calculated by analysing the entire sectional area of a large (> 2 cm^2^) lung tissue section. Pulmonary arteries and veins with a luminal diameter of less than 400 μm^2 ^were excluded from this analysis.

### Calculation of the Amount of Tissue in the Alveolar Parenchyma

The proportion of air (i.e. luminal spaces) to proper tissue in the alveolar parenchyma was determined after staining with Gomori's trichrome. From each patient, three regions of alveolar parenchyma without any visible bronchioles or vessels were analysed, each comprising an area of 1.20 mm^2^. After a threshold was selected for each parameter using the program ImageJ (NIH, Bethesda, MD) the proportion of air (i.e. luminal space) was calculated.

### Statistical Analysis

All analyses were done blinded and also counted by a second person. The mean values were used for the analysis. The variance between the results from the two observers were assessed by Bland-Altman plots including bias, calculation of the limits of agreement, and further calculation of intraclass correlation coefficients. For example, the combination CXCR4/prolyl 4-hydroxylase had a bias of 0.065, 95% limits of agreement from -5.2 to 5.4, and intraclass correlation coefficient of 0.98. The other combinations of markers to identify fibrocytes showed similar results. Results are shown as mean ± standard deviation. Differences between the patient and control groups regarding the number of fibrocytes, the percentage endothelia layer, the percentage lumen (of vessels), and percentage of tissue in alveolar parenchyma were analysed with the Mann-Whitney U test. All correlation analyses were done by using Spearman's rank correlation coefficient test. P-values of < 0.05 were considered significant.

## Results

### Patient Characteristics

**Figure 1:**
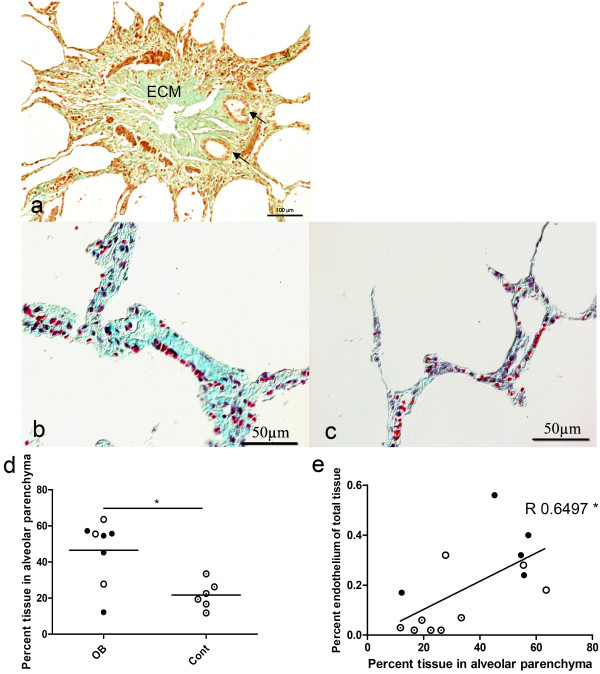
**Obliterative bronchiolitis patients had more compact alveolar parenchyma than the controls**. The structural changes in bronchioles were characterized by Gomori's trichrome staining. Panel *(a) *shows tissue from a lung-transplanted patient. The bronchiole has been obliterated with extra cellular matrix (ECM); the arrows show two neo-lumens. Scale bar: 100 μm; original magnification: 20×. Panels *b and c *show tissues in alveolar parenchyma from patients with thickened *(b) *and normal *(c) *alveolar parenchyma. Original magnification: 20×. Panel *d *shows changes in percentage tissue in alveolar parenchyma in the group of obliterative bronchiolitis patients. The amount of parenchyma was significantly greater in the obliterative bronchiolitis group than in the controls. Panel *e *represents the correlation between percentage area of the endothelial layer and percentage tissue in alveolar parenchyma (R = 0.6497; p < 0.05). Lung transplanted, closed circle; bone marrow transplanted, open circle; control, circle with dot.

Assessment of the general histopathology in Gomori's trichrome-stained sections revealed bronchioles with a narrowed lumen and characteristic fibrous scarring consistent with the diagnosis in the OB patients. This scarring consisted of ECM components such as collagen and proteoglycans (Figure 1a). In alveolar parenchyma, some patients had both thickening (Figure 1b) and normal parts (Figure 1c). The alveolar tissue was more dense in the OB patients 46.5 ± 17.6% than the controls 21.7 ± 7.6% (p < 0.05) (Figure 1d). There was a significant correlation between the total area of the endothelial layer and the tissue density in the alveolar parenchyma (R = 0.650 p < 0.05) (Figure 1e).

### Location of Pulmonary Fibrocytes

Regardless of the marker combination used to identify fibrocytes, these cells were found in varying numbers and localisations in the lung of both patient groups. Fibrocytes were present, either alone or in higher frequency, but without any cell-cell contact in the tissue.

**Figure 2:**
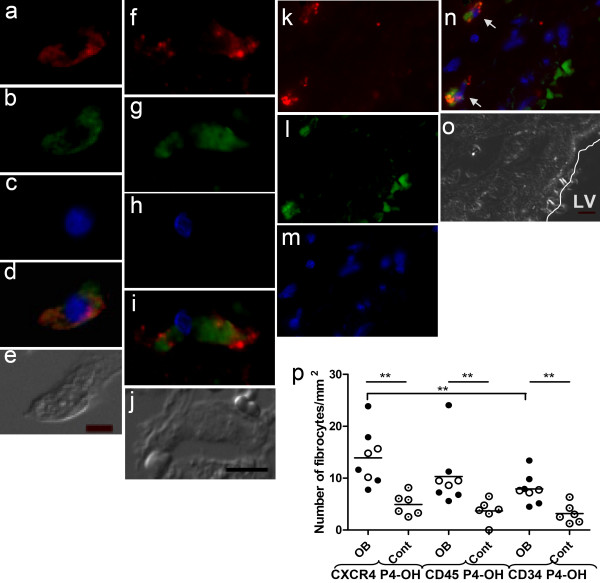
**Obliterative bronchiolitis (OB) patients had higher amounts of fibrocytes in lung tissue**. Fibrocytes were identified by fluorescence microscopy of lung tissues, by combining detection of one of the haematopoetic markers (CXCR4, CD45R0, or CD34) with detection of prolyl 4-hydroxylase. Panels *a-e *show a fibrocyte situated in the lumen of the alveolar structure. Panels *a*, *b*, and *c *show the separate markers for CXCR4, prolyl 4-hydroxylase, and nuclear staining, while panel *d *represents a merged image of the fibrocyte and panel *e *shows a differential interference contrast image. Panels *f-j *show a fibrocyte identified in parenchymal tissue: panels *f, g and h *show the separate markers for CD45R0, prolyl 4-hydroxylase, and nuclear staining, while panel *i *is a merged image of the fibrocyte and panel *j *is a differential interference-contrast image. Panels *k-o *show two fibrocytes (indicated with arrows), which were situated 32 μm and 45 μm from the endothelial layer. Panels *k-m *show the separate markers for CD34, prolyl 4-hydroxylase, and nuclear staining, while panel *n *is a merged picture of the fibrocyte and panel *o *is a differential interference-contrast image. Controls for unspecific binding of antibodies and background fluorescence were included in all experiments. Scale bars (10 μm) are indicated on the differential interference-contrast images. Original magnification: 40×. Panel *p *represents the numbers of fibrocytes identified in lung tissue from obliterative bronchiolitis patients and healthy controls. All three staining combinations showed significantly higher levels of fibrocytes in the obliterative bronchiolitis group as compared to the controls. In the obliterative bronchiolitis group, significantly more fibrocytes were stained with the detection of CXCR4 and prolyl 4-hydroxylase than with the detection of CD34 and prolyl 4-hydroxylase. Lumen of vessel is indicated as LV. Prolyl 4-hydroxylase is indicated as P4-OH. Lung transplanted, closed circle; bone marrow transplanted, open circle; control, circle with dot.

By using different staining combinations to identify subtypes of fibrocytes, the following results were obtained. When CXCR4 and prolyl 4-hydroxylase were detected, a fibrocyte was identified in the lumen closed to septa of the alveolar structure (Figure 2, a-e); when the combination of CD45R0 and prolyl 4-hydroxylase was examined, we identified fibrocytes in the septa of the alveolar parenchyma located 38 μm from a vessel (Figure 2, f-j). When CD34 and prolyl 4-hydroxylase were detected, fibrocytes close to (34 μm and 45 μm) the endothelial layer were seen (Figure 2, k-o).

### Fibrocytes Were Present at Elevated Levels in Patients with OB

In the OB patients, significantly higher numbers of lung fibrocytes were found (p < 0.01): 13.9 ± 5.27 fibrocytes/mm^2^, 10.3 ± 5.84 fibrocytes/mm^2^, and 7.91 ± 2.77 fibrocytes/mm^2 ^for CXCR4/prolyl 4-hydroxylase, CD45R0/prolyl 4-hydroxylase, and CD34/prolyl 4-hydroxylase, respectively, as compared to the controls: 4.92 ± 2.12 fibrocytes/mm^2^, 3.69 ± 2.14 fibrocytes/mm^2^, and 3.15 ± 1.89 fibrocytes/mm^2^. When the staining combinations were compared in the patient group, the staining combination of CXCR4 and prolyl 4-hydroxylase revealed significantly more fibrocytes than the combination of CD34 and prolyl 4-hydroxylase. Significantly higher numbers of fibrocytes were found in the LTP patients: 14.13 ± 6.64 fibrocytes/mm^2^, 10.99 ± 7.62 fibrocytes/mm^2^, and 8.15 ± 3.63 fibrocytes/mm^2 ^for CXCR4/prolyl 4-hydroxylase, CD45R0/prolyl 4-hydroxylase, and CD34/prolyl 4-hydroxylase, respectively, compared to the controls (p < 0.01, p < 0.01, and p < 0.05, respectively). Also, significantly higher numbers of fibrocytes were present in BMT patients: 13.54 ± 2.95 fibrocytes/mm^2^, 9.20 ± 0.50 fibrocytes/mm^2^, and 7.51 ± 0.28 fibrocytes/mm^2^, respectively, compared to the controls (p < 0.05 in all three comparisons) (Figure 2p).

### Patients with OB Have Remodelled Vessels

**Figure 3:**
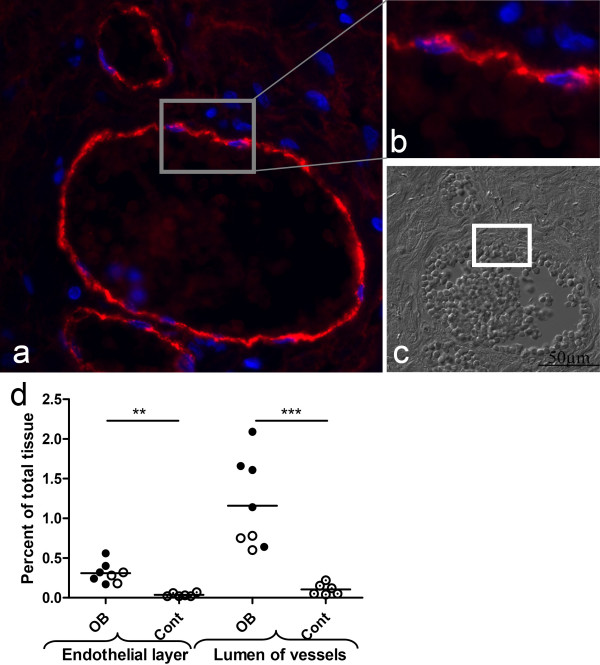
**Both the endothelial layer and the lumen of vessels were enlarged in obliterative bronchiolitis (OB) patients**. Vessels were stained with antibody to von Willebrand factor, and a characteristic vessel and a zoomed part of the endothelial layer of the vessel are shown in panels *a *and *b*, respectively. Panel *c *shows a differential interference contrast image from the same part of the tissue as in panel *a*. Scale bar (50 μm) is indicated on the differential interference-contrast image. Original magnification: 20×. The total area of the endothelial layer was significantly enlarged in the patient group compared to the controls. The total area of the lumen of the vessels was significantly enlarged in the patient group compared to the controls. Lung transplanted, closed circle; bone marrow transplanted, open circle; control, circle with dot.

There was a significantly higher proportion of the endothelial layer in the patients with OB (0.31 ± 0.13%) than in the controls (0.037 ± 0.02%) (p < 0.01). The LTP subjects had significantly greater total area of the endothelial layer (0.34 ± 0.15%) than the controls (p < 0.01) and the corresponding significance level for BMT was 0.05 with (0.26 ± 0.07%) of total tissue being endothelial layer. The percentage area of the lumen in the vessels was significantly greater in the patients with OB (1.16 ± 0.57%) than in the controls (0.10 ± 0.07%) (p < 0.001). LTP patients alone had significantly larger percentage area of the lumen (1.43 ± 0.55%) than the controls (p < 0.01). The BMT patients also had significantly larger total area of the lumen (0.71 ± 0.10%) than the controls (p < 0.05) (Figure 3a-d).

### Correlation between Tissue Remodelling and Number of Fibrocytes

**Figure 4:**
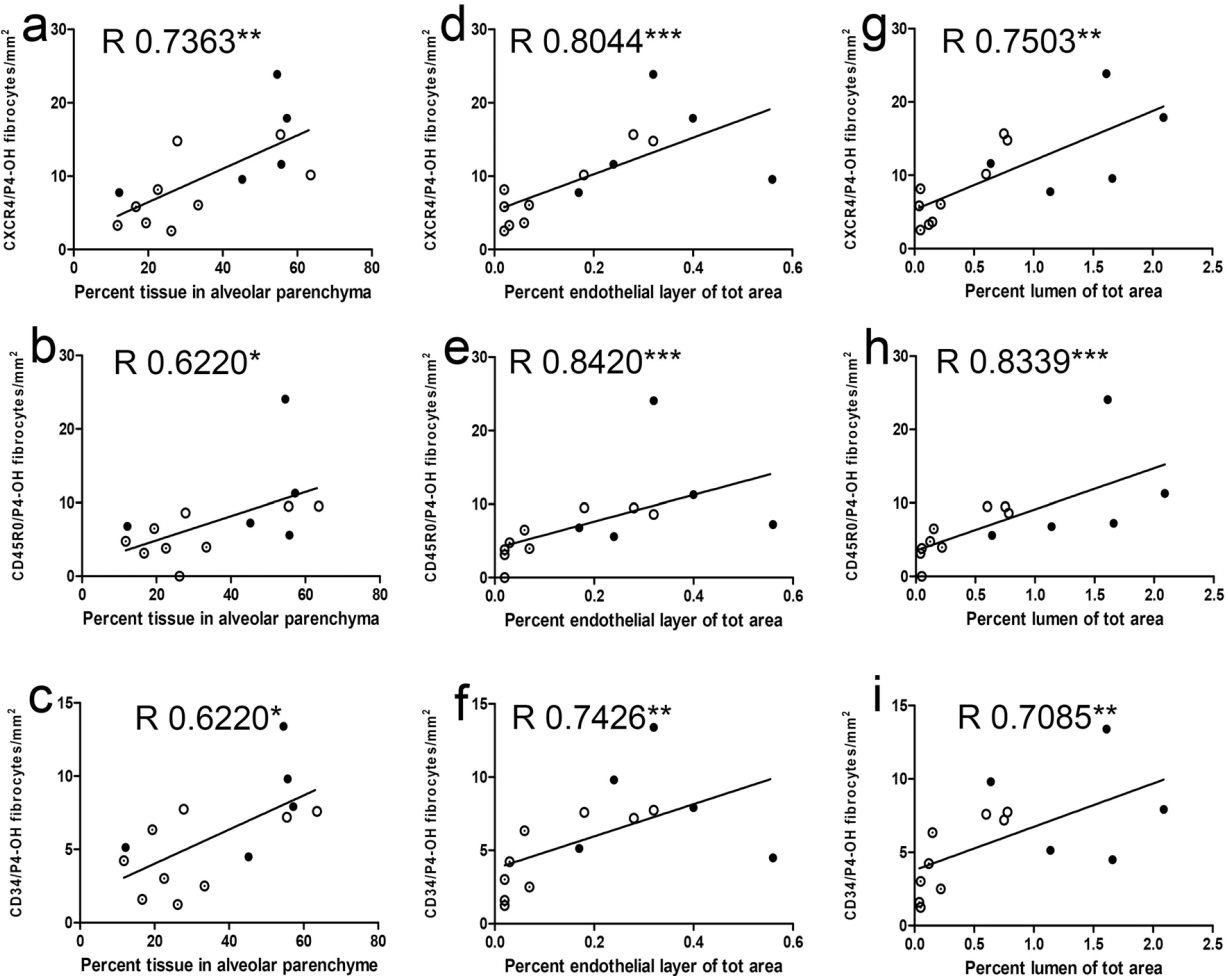
**There was a correlation between numbers of fibrocytes and percentage of tissue in alveolar parenchyme, total area of the endothelial layer, and total area of the lumen**. Panels *a-c *represent correlations between percentage of tissue in alveolar parenchyma and number of fibrocytes stained with markers CXCR4 and prolyl 4-hydroxylase (R = 0.7363; p < 0.01) *(a)*, CD45R0 and prolyl 4-hydroxylase (R = 0.6220; p < 0.05) *(b)*, and CD34 and prolyl 4-hydroxylase (R = 0.6220; p < 0.05) *(c)*. Panels *d-f *represent correlations between percentage endothelial layer (of total area of tissue) and number of fibrocytes stained with markers CXCR4 and prolyl 4-hydroxylase (R = 0.8044; p < 0.001) *(d)*, CD45R0 and prolyl 4-hydroxylase (R = 0.8420; p < 0.001) *(e)*, and CD34 and prolyl 4-hydroxylase (R = 0.7426; p < 0.01) *(f)*. Panels *g-i *represent correlations between percentage vessel lumen (of total area of tissue) and number of fibrocytes stained with markers CXCR4 and prolyl 4-hydroxylase (R = 0.7503; p < 0.01) *(g)*, CD45R0 and prolyl 4-hydroxylase (R = 0.8339; p < 0.001) (*h*), and CD34 and prolyl 4-hydroxylase (R = 0.7085; p < 0.01) *(i)*. Prolyl 4-hydroxylase is indicated as P4-OH. Lung transplanted, closed circle; bone marrow transplanted, open circle; control, circle with dot.

The numbers of fibrocytes identified with the three combinations of specific markers correlated well with the amount of alveolar parenchyma: CXCR4/prolyl 4-hydroxylase (Figure 4a); CD45R0/prolyl 4-hydroxylase (Figure 4b); and CD34/prolyl 4-hydroxylase (Figure 4c). Furthermore, there were also correlations between the numbers of fibrocytes and the total areas of the endothelial layer and total areas of the lumen: CXCR4/prolyl 4-hydroxylase (Figure 4d); CD45R0/prolyl 4-hydroxylase (Figure 4e); and CD34/prolyl 4-hydroxylase (Figure 4f). The same patterns were seen in the correlation between total area of the lumen and number of fibrocytes: CXCR4/prolyl 4-hydroxylase (Figure 4g); CD45R0/prolyl 4-hydroxylase (Figure 4h); and CD34/prolyl 4-hydroxylase (Figure 4i).

### Endothelium Layer Change with Time from Transplantation to Autopsy

**Figure 5:**
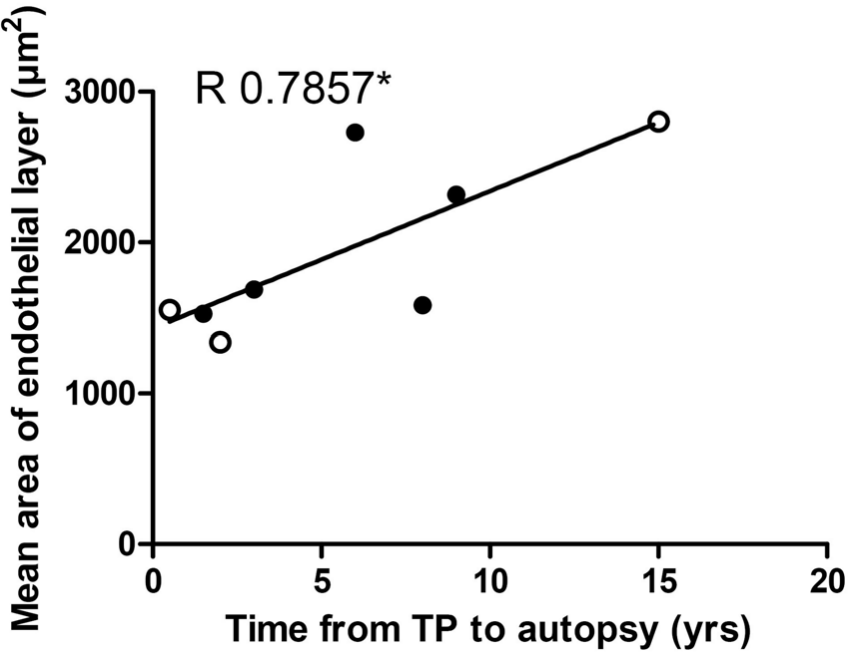
**Endothelial layer increases with time from transplantation to autopsy**. The figure represents the correlation (R = 0.7857; p < 0.05) between mean area of the endothelial layer and time from transplantation to autopsy in patients with obliterative bronchiolitis. Lung transplanted, closed circle; bone marrow transplanted, open circle.

In addition, there was a correlation between the mean area of the endothelial layer and the time from transplantation to autopsy in patients with OB (Figure 5).

### Number of CD34/prolyl 4-hydroxylase fibrocytes increase with age at autopsy

**Figure 6:**
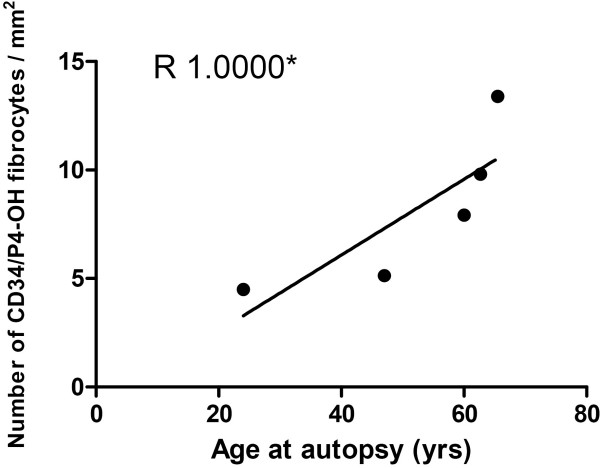
**Number of CD34/prolyl 4-hydroxylase fibrocytes increase with age at autopsy**. The figure represent a correlation between the number of CD34/prolyl 4-hydroxylase fibrocytes and the age of the lung transplanted patients with OB at time of autopsy R = 1.00 (p < 0.05) (Figure 6).

Moreover, there was a correlation between the number of CD34/prolyl 4-hydroxylase fibrocytes and the age of the lung transplanted patients with OB at time of autopsy (Figure 6).

## Discussion

This study shows a correlation between structural remodelling of several compartments of the lung and the number of fibrocytes identified in tissue from patients with OB after transplantation. The fibrocytes were identified in both the patient and the control groups by combining detection of different markers (CXCR4/CD34/CD45R0) with detection of prolyl 4-hydroxylase. However, more fibrocytes were identified in the patient group - and especially in the LTP patients - than in the controls. The combination CXCR4 together with prolyl 4-hydroxylase identified the highest number of fibrocytes relative to the other combinations and the patient group also had a larger proportion of endothelial layer combined with a larger amount of vessel lumen compared to the controls. The number of fibrocytes in lung tissue was also correlated to remodeling changes of the pulmonary vessels. Moreover, there was a gradual increase in endothelial layer seen over time from transplantation to autopsy.

There is currently a debate as to whether fibrocytes are good or bad in airway remodelling. Recently, however, various studies have indicated that there is an association between the occurrence of fibrocytes and pathological structural changes of the airways, paralleled by a decline in lung function. In idiopathic pulmonary fibrosis, it has been demonstrated that there is a correlation between the number of fibrocytes lung tissue and the number of fibroblastic foci [14]. Also, Moeller *et al. *have shown that the number of circulating fibrocytes is elevated during an exacerbation and that the number of these cells may be useful as a predictor of early mortality in patients with IPF [20]. Furthermore, Nihlberg *et al. *have demonstrated that in asthma patients basement membrane thickness may be correlated to the number of fibrocytes in lung tissue [13]. Also other studies have shown who the number of circulated fibrocytes are increased by severity of the disease [21,22]. In accordance with this, the present study has shown a correlation between the number of fibrocytes and degree of structural remodeling of the alveolar parenchyma as well as degree of remodeling of the vessels. These findings indicate that the fibrocytes have a negative effect, at least at the end-stage of OB. Fibrocytes appear to indicate an ongoing chronic reparative process, at least in the pulmonary diseases described.

It can be argued that the correlation studies are of less value as there is already a significant difference between the control- and patient group in number of fibrocytes and structural related changes of the tissue. Nevertheless, the patient material consists of a mixed population of various disease states (Table 1) with several interlaced values between lowest and highest points of the correlation line, indicating true correlations.

Fibrocytes could contribute this chronic reparative process in several ways. When the fibrocytes are leaving the circulation and passing through the vascular barrier into tissue, they are able to secrete a cascade of factors: pro-angiogenic factors such as vascular endothelial growth factor (VEGF) and haematopoietic growth factors that can change the surrounding environment and the vascular bed by promoting endothelial cell migration, proliferation, and/or tube formation [23]. Fibrocytes are also able to produce ECM and to differentiate into fibroblasts or myofibroblasts [24], which are the main producers of both collagen and proteoglycans such as decorin, versican, and biglycan [25], and thus could contribute to the development of the fibrotic lesions seen in OB. This differentiation is possible when the fibrocyte has entered the tissue after rolling. Vessel changes in the bronchial circulation occur in lung diseases such as asthma [26], but also in patients with OB [18]. The remodelling of the pulmonary circulation has not been fully investigated. For example, in IPF the changes depend both on the progress of the fibrosis and on the distance from fibroblastic foci, with more vessels early in the disease and outside fibroblastic foci [27]. In the present study, all the patients were at the end-stage of the disease and the remodelling process had continued over time.

The group of patients who had undergone BMT seemed to be more homogenous than the LTP group, both regarding the number of fibrocytes and the degree of remodelling of vessels. This may be explained by the age of the patients. All the patients had a close age range - between 10 and 15 years of age at the time of BMT and between 12 and 30 years old when they were lung transplanted. There was a positive correlation between the number of fibrocytes identified by CD34 and prolyl 4-hydroxylase and age at the time of autopsy in LTP patients. Our findings are further supported by a study by Mora *et al. *that showed that the number of circulating fibrocytes in mice varied with the age of the mouse, with a higher number being found in the older animals [28].

It has been reported that patients with OB after LTP have elevated levels of transforming growth factor beta (TGF-β) in respiratory epithelial lining fluid and that cells from bronchoalveolar lavage show increased expression of TGF-β [29]. Even though the level of TGF-β in tissue has only been evaluated in animal models of chronic allograft rejection [30], it has been shown that TGF-β is a central cytokine in the development of fibrosis [31]. Furthermore, the expression of VEGF and haematopoietic growth factors can regulate the TGF-β-dependent development of fibrotic lesions [32].

A potentially important agent of both fibrocyte differentiation and of the remodeling of vessels is therefore TGF-β. This cytokine has been shown to differentiate blood-derived fibrocytes to αSMA-expressing myofibroblasts [22]; in addition, it has also been shown to increase the expression of certain ECM molecules that could function in stabilising vessels.

It is hypothesised that hypoxia is a major contributor to the induction of remodelling of vessels and in the balance between deposition and degradation of ECM. This could occur due to an increased level of nitric oxide which would result in vasodilation, increased vasopermeability, and initiation of angiogenesis while the hypoxia itself would increase expression of VEGFR. Hypoxia is a well-characterized condition, which elicits expression of hypoxia-induced factor (HIF) [33], SDF-1 [34], and CXCR4 [35] and may give rise to recruitment of progenitor cells such as fibrocytes. HIF can upregulate expression of prolyl 4-hydroxylase [36], a key enzyme in the biosynthesis of collagen, and thus more collagen and ECM would be synthesised and accumulated. It has also been shown that SDF-1 can bind to ECM molecules [37], where the ECM can function as a reservoir and also help to sustain the fibrotic reaction. SDF-1 can also be released from the ECM under specific conditions such as injury or stress.

In summary, these novel findings have provided us with some insight into the complexity of OB. Both the remodelling of vessels and the thickening of the alveolar parenchyma are related to the number of fibrocytes, which can be assumed to have an important role in OB. Future studies of these cells will help us to understand the mechanisms behind this very serious complication of transplantation. To further increase the understanding about the fibrogenesis in OB, complementary studies on additive effects of EMT and resident fibroblast on this process are warranted.

## Competing interests

The authors declare that they have no competing interests.

## Authors' contributions

All authors have read and approved the final manuscript. AAS: Participated in the design of the study, analysis and interpretation of data, performed the statistical analysis, and drafted the manuscript. JE: Revising the manuscript critically for important intellectual content. LB: Revising the manuscript critically for important intellectual content. LE: Participated in the design of the study and revising the manuscript critically for important intellectual content. GWT: Participated in the design of the study and revising the manuscript critically for important intellectual content.
